# Exploration of the Molecular Mechanism of Danzhi Xiaoyao Powder in Endometrial Cancer through Network Pharmacology

**DOI:** 10.1155/2022/8330926

**Published:** 2022-06-13

**Authors:** Lanyu Li, Lukai Yang, Fang Liu, Jinfeng Qu

**Affiliations:** ^1^Department of Obstetrics and Gynecology, Central Hospital Affiliated to Shandong First Medical University, Jinan 250013, China; ^2^Department of Gastroenterology & GI Endoscopy Center, Dongying People's Hospital, Dongying 257091, China

## Abstract

Endometrial cancer (EC) is a common malignant tumor of the female reproductive system. Current treatments such as surgery and long-term hormone therapy are ineffective and have side effects. Danzhi Xiaoyao powder (DXP) can inhibit the growth of EC cells and induce apoptosis, but the pharmacological and molecular mechanisms of anticancer effects are still unclear. In this study, active components and potential targets of DXP were obtained from public databases. Protein effects and regulatory pathways of common targets were analyzed by protein-protein interaction (PPI), GO and KEGG. The results of network pharmacology showed that there are 87 common targets between EC and DXP. GO enrichment analysis showed that these targets were associated with response to oxidative stress, response to nutrient levels, hormone receptor binding and nuclear hormone receptor binding, etc. The results of KEGG analysis indicated that IL-17, TNF, PI3K/AKT, and RAS/RAF/MEK/ERK (ERK) signaling pathway were enriched in the anti-EC of DXP. Additionally, we cultured HEC-1B and KLE cells for validate experiments. DXP showed an inhibition of proliferation, migration, and cell cycle of both cells. Moreover, the expression of RAS, p-RAF, p-MEK, ERK, and p-ERK related proteins were downregulated. In conclusion, DXP might inhibit the proliferation of EC cells via apoptosis. Furthermore, DXP-induced inhibition of EC development might involve RAS/RAF/MEK/ERK pathway.

## 1. Introduction

Endometrial cancer (EC) is one of the malignant tumors of the female reproductive system, and its prevalence and mortality have rapidly risen worldwide [1, 2]. Globally, there were an estimated 417,000 incident cases and 97,000 deaths in 2020 [3]. The occurrence of EC is usually associated with overexposure to endogenous or exogenous estrogens [4]. Currently, the main treatment methods for EC are surgery and chemotherapy [5]. Chemotherapy drugs have certain toxicity, and due to the evolutionary nature of tumors, tumors cells may differentiate into subclonal cells with certain drug resistance, resulting in reduced antitumor efficacy of these chemotherapy drugs and possible adverse reactions [6, 7]. Therefore, it is important to find drugs with low side effects and better efficacy to improve the condition of EC.

With the development of cellular biotechnology, breakthroughs have been made in the research of anticancer herbal medicines. In the past 20 years, natural antitumor drugs have accounted for 66.29% of the anticancer drugs on the market [8]. Traditional Chinese medicine (TCM) has unique advantages in terms of efficacy and safety. Its reasonable application can help control the development of the disease, reduce the toxic effect of radiotherapy and avoid the recurrence of cancer metastasis to a large extent [9]. Danzhi Xiaoyao powder (DXP) is a formula of TCM including *Cortex Moutan, Gardeniae Fructus, Radix Bupleuri, Angelicae Sinensis Radix, Paeoniae Radix Alba, Atractylodes macrocephala Koidz., Poria Cocos (Schw.) Wolf., and Licorice* [10]. Studies have found that DXP has antidepressant [11] effects, and some studies have found that DXP has some efficacy against breast cancer and precancerous lesions [12, 13], especially ER-positive breast cancer [13], suggesting that DXP may also have some anticancer effects. Clinical studies have shown that DXP has good therapeutic effects on patients with endometriosis liver depression and blood heat type [14]. Therefore, DXP has the potential to be a drug for the treatment of EC. However, its active components, possible targets, and molecular mechanism have not been elucidated.

TCM plays a therapeutic role by restoring homeostasis in the biological process during disease development through multiple targets [15]. Network pharmacology is a new method to analyze the relationship between drugs, ingredients, targets, and diseases in the network based on biological networks and is gaining importance [16–18]. Therefore, the combining of TCM and network pharmacology is a new approach for the preferential selection of disease-related genes, revealing the association between diseases and drugs and further elucidating the regulatory role of TCM [19, 20].

In this study, network pharmacology combined with *in vitro* cellular experiment validation was performed to explore the active components, potential targets, and molecular mechanism of the anti-EC effect of DXP.

## 2. Materials and Methods

### 2.1. Collection of EC-Related Targets

We searched the Online Mendelian Inheritance in Man (OMIM, https://www.omim.org/) [21], DisGeNET (https://www.disgenet.org/) and GeneCards (https://www.genecards.org/) with the key word “endometrial cancer” to obtain EC-related targets. The duplicate disease targets were merged and deleted.

### 2.2. Collection of Active Components and Potential Targets of DXP

To collect all the relative components of DXP, we searched the traditional Chinese medicine systems pharmacology database and analysis platform (TCMSP, https://lsp.nwu.edu.cn/tcmsp.php) [22], Traditional Chinese Medicine Integrated Database (TCMID, https://tcm.cz3.nus.edu.sg/group/tcm-id/tcmid) [23], and Herbal Ingredients' Targets Database (HIT, https://lifecenter.sgst.cn/hit) [24], which includes a great deal of information about Chinese herbal medicine. All predicted targets of active components of DXP were searched from TCM-ID, TCMSP, HIT, and Search Tool for Interacting Chemicals (STITCH, https://stitch.embl.de) databases and selected according to the component-target correlation degree above 400 from STITCH database.

#### 2.2.1. Drug-Likeness Screening

In the early stages of drug discovery, drug-likeness is a key consideration in the selection of compounds. A quantitative estimate of drug-likeness (QED), reported by Bickerton et al. in 2012, was used to evaluate a component's drug-likeness [25]. In this work, DXP components with QED ≥ 0.3 were included for further study.

#### 2.2.2. Binomial Statistical Mode Analysis

According to previous studies, a binomial statistical mode was used for secondary screening for active components and potential targets of DXP [26, 27]. In this study, we set targets with *P* < 0.05 that could be used as main targets for the formula.

### 2.3. Common Target Screening and Protein-Protein Interaction (PPI) Construction

The gene targets of components of DXP and EC were analyzed by Venny (version 2.1) to obtain the common genes, which were the targets of action of DXP for the treatment of EC.

The PPI network was constructed by importing the common targets to the String database (https://string-db.org/cgi/input.pl), limiting the species to “Homo sapiens.” The results were imported into Cytoscape (version 3.8.0) for further analysis and optimization of the protein interaction network.

### 2.4. Construction of Herb-Components-Target (H-C-T) Network

An H-C-T network was constructed based on the active components of DXP and common targets. The above information was imported into Cytoscape for further analysis.

### 2.5. Enrichment Analysis

The clusterProfiler package of R (version 3.15.4) was applied for Gene Ontology (GO) and Kyoto Encyclopedia of Genes and Genomes (KEGG) analysis. The GO analysis enriching for 3 aspects of potential targets is as follows: biological process (BP), cellular component (CC), and molecular function (MF). *P* < 0.01 was set as a screening threshold and the functional terms and pathways with this value were considered statistically significant and retained.

### 2.6. Cell Culture

The EC cell line HEC-1B and KLE cells were purchased from the Shanghai Institute for Biological Sciences, Chinese Academy of Sciences. HEC-1B cells were cultured in Dulbecco's Modified Eagle's Medium (DMEM) medium (Gibco, MD, USA) with 10% fetal bovine serum (Gibco); the KLE cells were cultured in DMEM/F12 medium (Gibco) with 10% fetal bovine serum (Gibco), both at 37°C with 95% air and 5% CO_2_.

### 2.7. Detection of Cell Viability

HEC-1B and KLE cells (2 × 10^3^/well) were seeded into 96-well plates and cultured overnight at 37°C with 95% air and 5% CO_2_. Then, cells were treated with different concentrations of DXP (0, 0.05, 0.1, 0.2, 0.4, 0.8, and 1.6 mg/mL) for 24 h and 48 h. Next, cells were incubated with CCK-8 solution (Biosharp, China) for 2 h at 37°C and the absorbance at 450 nm was measured by an automatic microplate reader (BioTek, VT, USA).

### 2.8. Flow Cytometry

HEC-1B and KLE cells were cultured in 6-well plates at 3 × 10^5^ cells/well and treated with 0.4 or 0.8 mg/mL DXP for 48 h, respectively.

For cell cycle analysis, cells were collected and washed with phosphate-buffered saline (PBS) followed by prechilled 70% ethanol at 4°C for more than 4 h. After fixation, cells were incubated with 100 *μ*g/mL RNase (Nanjing KeyGen Biotech Co., Ltd., China) for 30 min, followed by 50 *μ*g/mL propidium iodide (PI) (Becton-Dickinson, NJ, USA) for 30 min in the dark. Finally, a flow cytometer (Becton-Dickinson) was used to detect the cell cycle of HEC-1B and KLE cells.

For apoptosis analysis, cells were digested with trypsin without EDTA, washed twice with prechilled PBS, and then resuspended in the binding buffer. Next, cells were incubated with Annexin V-FITC (Becton-Dickinson) and PI for 15 min at room temperature under light-proof conditions. Finally, a flow cytometer (Becton-Dickinson) was used to detect the cell apoptosis of HEC-1B and KLE cells.

### 2.9. Cell Migration

The ability of migration in HEC-1B and KLE cells was detected by transwell assays. HEC-1B and KLE cells were resuspended in serum-free medium. 200 *μ*L·cell suspensions were added to the upper layer of the transwell chamber (Corning, NY, USA). The lower chamber contained 500 *μ*L of serum-containing medium. After 48 h incubation, the cells in the lower chamber were stained with 0.1% crystal violet for 20 min and counted under a 400 × microscope (Olympus, Japan) in 5 random fields.

### 2.10. Western Blot

The cells were collected and lysed with cell lysis buffer (Beyotime, China) for 30 minutes on ice, then centrifuged at 12000 rpm and 4°C for 10 minutes. The supernatants were collected and the protein concentrations were quantified by the BCA kit (Pierce, IL, USA). The protein samples were separated by the 10% SDS-PAGE gradient gel. The separated protein was transferred onto PVDF membranes. The membranes were blocked with 5% skim milk for 90 min, and incubated with primary antibodies (1 : 1000, Abcam, UK) overnight at 4°C. Following this, they were incubated with secondary antibody (1 : 500,Hangzhou Multi Sciences Co., Ltd., China, GAR007) for 1 h. Finally, the protein bands were detected by an enhanced chemiluminescent (ECL) system (Beyotime). The primary antibodies were listed as below: RAS (ab52939), p-RAF (ab200653), MEK1 (ab32091), p-MEK1 (ab96379), ERK (ab32537), p-ERK (ab131438), and GAPDH (ab9485).

### 2.11. Statistical Analysis

The results were presented as means ± SD and data comparison of multiple groups was adopted by one-way ANOVA followed by Tukey's test. All statistical analyses were obtained by GraghPad 7.0. The results were considered statistically significant if the *P* value was less than 0.05.

## 3. Results

### 3.1. EC-Related Targets

By retrieving three public databases, 836 EC-related targets were obtained, which included 237 in GeneCards, 197 in OMIM, and 402 in DisGeNET with the key word “endometrial cancer.” A total of 679 targets were screened by combining and deleting the repetitive values.

### 3.2. Active Components and Potential Targets of DXP

**Figure 1:**
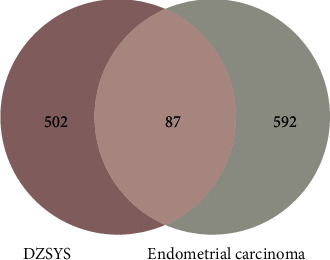
Venn diagram of common targets of danzhi xiaoyao powder (DXP) and endometrial cancer (EC).

A total of 321 DXP-related components were searched from public databases including HIT, TCMSP, and TCMID databases. The potential targets of DXP were obtained from four public databases (HIT, TCMSP, TCMID, and STITCH), and there were 5,558 targets related to active components of DXP. Then, 274 active components were obtained according to drug-likeness screening (QED ≥ 0.3). Moreover, 589 main targets were obtained from binomial statistical mode analysis. Following it, we retrieved 214 active components of DXP. Among 589 screened components-related targets and 679 EC-related targets, 87 common targets were identified and considered as the key targets for further study (Figure 1). Accordingly, 168 active components were screened and identified as the main active components of DXP that act on EC (Table 1).

### 3.3. Construction of PPI Network

**Figure 2:**
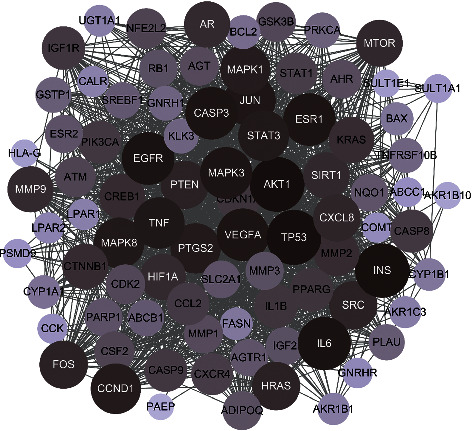
Common target protein-protein interaction (PPI) network between DXP and EC. Each node represents a target. The lines between the nodes represent the association among the different targets. The size and color of the node and its color shade change represent the size of the degree value.

The 87 common targets of DXP and EC were imported to the String database for analysis, and Cytoscape (version 3.8.0) was used for PPI construction (Figure 2). It contained 87 nodes and 1,587 edges, and the average degree value was 36.5.

### 3.4. Construction of Herb-Components-Target (H-C-T) Network

**Figure 3:**
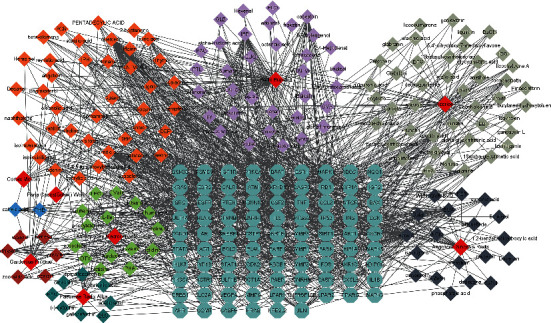
Herb-components-target (H-C-T) network. The red rhombus represents the different herbs contained in DXP. The cyan hexagon represents the key targets of DXP acting on EC. Other different colors rhombuses represent the compounds contained in different herbs. Among them, the orange rhombus represents the components that are contained in many kinds of herbs.

The H-C-T network was obtained through the import of 168 active components and 87 potential common targets of DXP into Cytoscape (version 3.8.0) and is shown in Figure 3. There were 262 nodes and 1159 edges. The node size was proportional to its degree value. The red rhombus stood for different herbs which were contained in DXP. The cyan hexagon represented key targets of DXP acting on EC. Other different colored rhombuses represented the compounds contained in different herbs. Among them, the orange rhombus stood for the components that were contained in many kinds of herbs. The result showed that the top 5 active components nodes with the largest size of degree were quercetin, ethanol, kaempferol, IFP, and palmitic acid, with degree values of 64, 52, 43, 42, and 41, respectively. CASP3, AKT1, CYP1A1, JUN, and PPARG exhibited the highest degree values of 48, 25, 24, 22, and 22, respectively. These active compounds and targets may play a vital role in the effect of DXP against EC.

### 3.5. Enrichment Analysis

**Figure 4:**
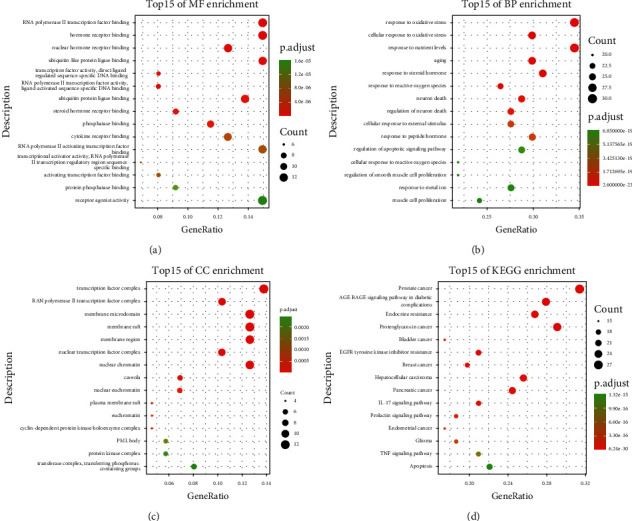
Enrichment analysis of GO and KEGG pathways of key target genes. The size of the circle represents the number of genes, and the color of the circle represents the corrected *P* value.

In order to illuminate the biological functions and signaling pathways during the process of anti-EC of DXP, we employed the clusterProfiler package of the R language to perform GO and KEGG enrichment analysis on the 87 targets. A total of significant GO 1123 terms were obtained, including 1066 terms of BP, 18 terms of CC, and 39 terms of MF (*P* < 0.01). The top 15 enriched terms of BP, CC, and MF were selected and visualized. The results showed that the targets were related to response to oxidative stress, response to nutrient levels, response to steroid hormone, and other biological processes; enriched in the transcription factor complex, membrane raft, membrane microdomain, and other cellular components; and related to RNA polymerase II transcription factor binding, hormone receptor binding, ubiquitin-like protein ligase binding, and other molecular functions (Figures 4(a)–4(c)).

**Figure 5:**
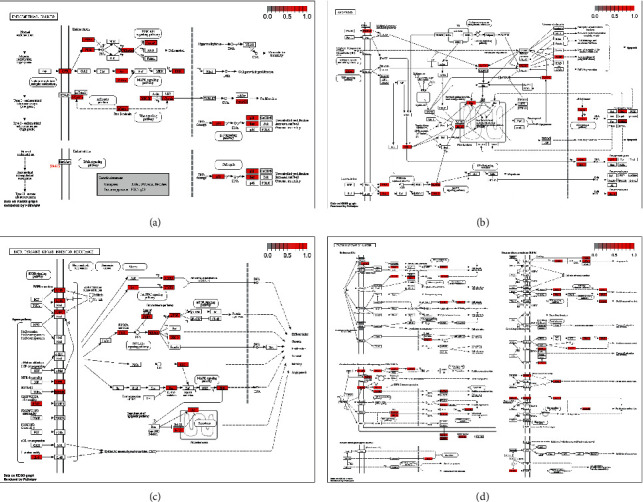
The map of endometrial cancer (a), apoptosis (b), EGFR tyrosine kinase inhibitor resistance (c), and proteoglycans in cancer (d) signaling pathway. The red boxes represent key targets.

In KEGG enrichment analysis, a total of 143 pathways of anti-EC targets of DXP were identified according to the *P* value less than 0.01. The top 15 significantly enriched pathways with the highest gene counts were shown in Figure 4(d), and the specific information was shown in Table 2. The results showed that the targets may be associated with the AGE-RAGE signaling pathway in diabetic complications, the IL-17 signaling pathway, the TNF signaling pathway, and other signaling pathways. Among them, the endometrial cancer, apoptosis, EGFR tyrosine kinase inhibitor resistance, and proteoglycans in the cancer signaling pathway map showed that the PI3K-AKT and RAS-RAF-MEK-ERK (ERK) signaling pathways may play a crucial role in the treatment of EC with DXP (Figure 5).

**Figure 6:**
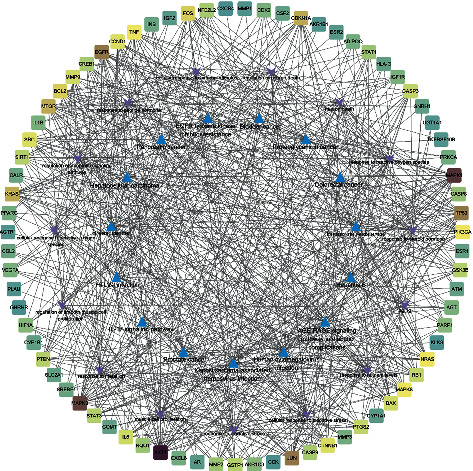
Core target-biological process-signal pathway network. The square represents DXP anti-EC targets, the triangle represents the top 15 signaling pathway, and the arrow represents the top 15 biological processes.

The network of target-BP-pathways was obtained by Cytoscape (version 3.8.0) and shown in Figure 6, which contained 105 nodes and 713 edges and included 75 common targets, the top 15 BP terms, and the top 15 signaling pathways. The square stood for disease targets, the triangle stood for signaling pathway, and the arrow represented biological processes.

### 3.6. Correlation Analysis of Targets with BP and Signaling Pathways

**Figure 7:**
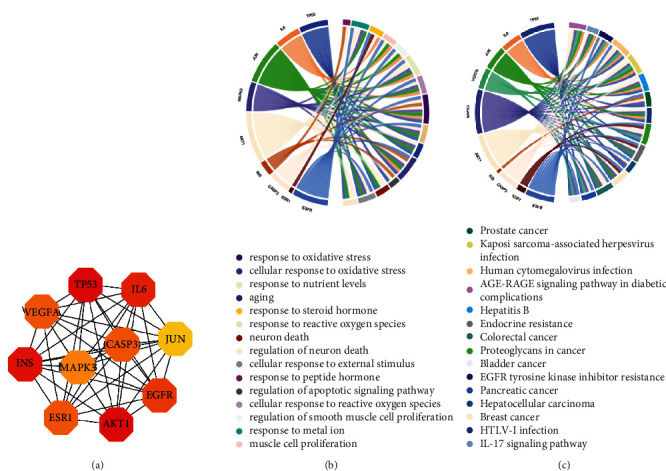
Hub network of top 10 high-degree targets (a), the correlation between the core network and the first 15 BP terms (b), and the correlation between the core network and the first pathway (c).

To explore the correlation of the hub targets with the signaling pathway and BP more clearly, a hub target network was constituted with the top 10 targets, including TP53 (*n* = 71), AKT1 (*n* = 71), INS (*n* = 70), IL6 (*n* = 67), EGFR (*n* = 66), CASP3 (*n* = 65), ESR1 (*n* = 65), VEGFA (*n* = 65), MAPK3 (*n* = 64), and JUN (*n* = 63) (Figure 7(a)). The correlations were analyzed by combining them with the top 15 pathways and BP terms. The results showed that AKT1 had a higher correlation with the BP, and its response to oxidative stress was more relevant to the targets (Figure 7(b)). In the analysis of correlation with signaling pathways, AKT1 and MAPK3 were more important to the pathways (Figure 7(c)).

### 3.7. DXP Suppresses the Viability, Cell Cycle, and Metastasis While Triggers the Apoptosis of EC Cells

**Figure 8:**
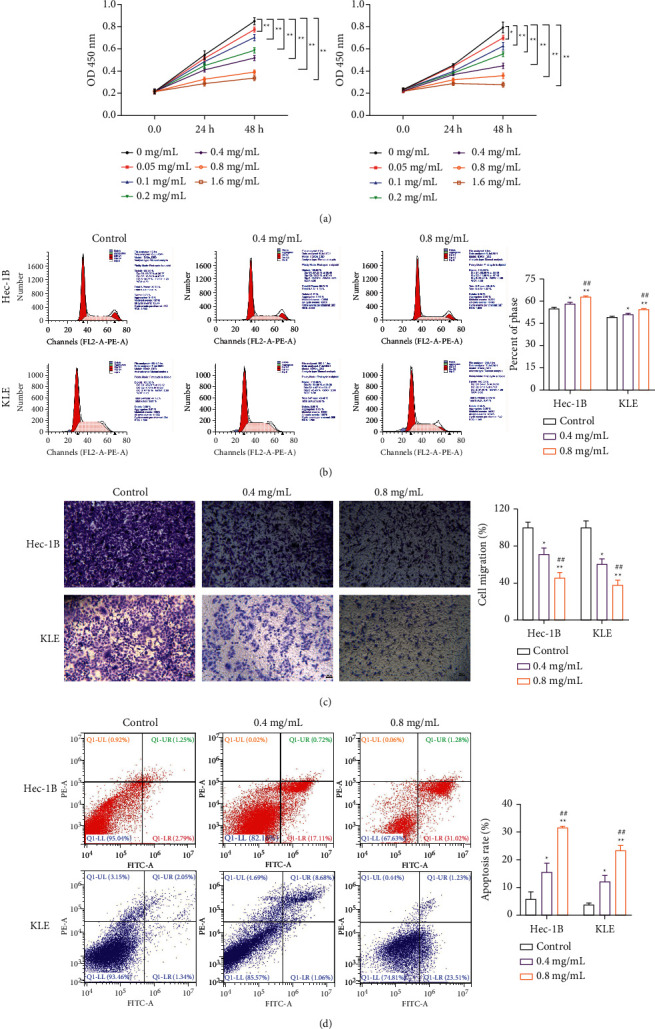
HEC-1B and KLE cells were treated with different concentrations of DXP for cell proliferation (a), cycle (b), migration (c), and apoptosis (d) assays. ^*∗*^*P* < 0.05, ^*∗∗*^*P* < 0.01 vs. Control group; ^##^*P* < 0.01 vs. 0.4 mg/mL group.

Different concentrations of DXP were used to treat HEC-1B and KLE cells and assay their survival rates. As shown in Figure 8(a), the cell viabilities of both types were decreased with the increasing concentration of the DXP at 24 h and 48 h. The LC_50_ values were calculated to be 0.717 mg/mL for HEC-1B and 0.603 mg/mL for KLE when DXP treated for 48 h. So, DXP (0.4 mg/mL and 0.8 mg/mL) treated HEC-1B and KLE cells for 48 h were selected in the subsequent experimental.

Cell cycles results showed DXP significantly increased the proportion of G0/G1 phase cells (*P* < 0.05), and DXP with a high-dose have a better inhibitory effect than low-dose (*P* < 0.01) (Figure 8(b)). Meanwhile, DXP also inhibited the migration of EC cells. The apoptosis rate in high-dose was significantly higher than low-dose (*P* < 0.01) (Figure 8(c)). Additionally, the apoptosis rate of EC cells was promoted by DXP addition (*P* < 0.05) (Figure 8(d)). These results indicated that DXP suppressed the malignant progress of EC cells *in vitro*.

### 3.8. DXP Inhibited the Protein Expression of ERK Signaling Pathway

**Figure 9:**
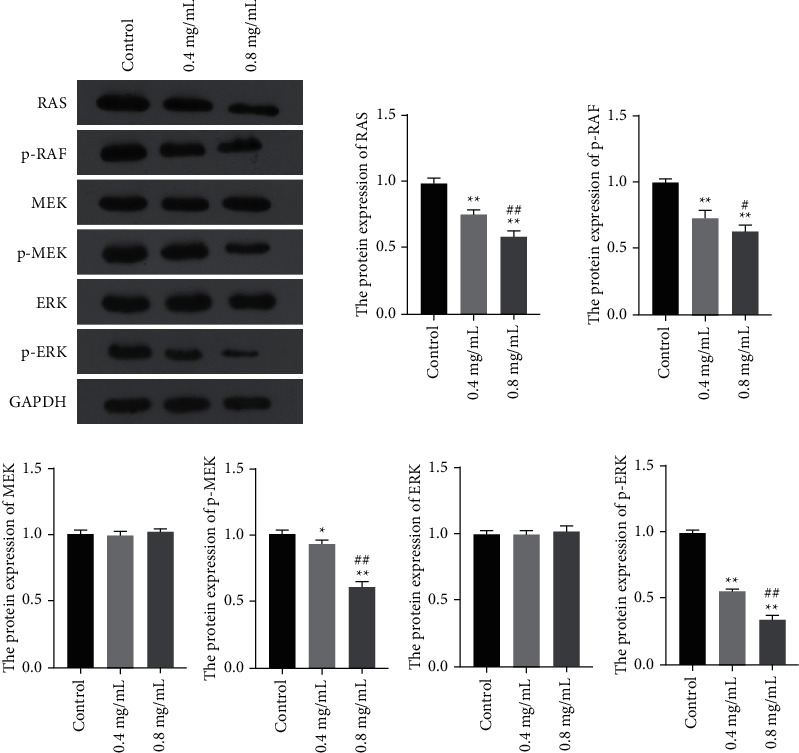
The expression of RAS/RAF/MEK/ERK (ERK) pathway related proteins was detected using Western blot. ^*∗*^*P* < 0.05, ^*∗∗*^*P* < 0.01 vs. Control group; ^#^*P* < 0.05, ^##^*P* < 0.01 vs. 0.4 mg/mL group.

Network pharmacology analysis indicated that the RAS pathway is the most important pathway in the treatment of DXP on EC. Thus, the expression levels of RAS, p-RAF, MEK, p-MEK, ERK, and p-ERK were examined by Western blot. As shown in Figure 9, DXP decreased the expression of RAS, p-RAF, p-MEK, and p-ERK proteins, but had no effect on MEK and ERK protein expression. It suggested that DXP against EC through ERK signaling pathway.

## 4. Discussion

As an epithelial malignant tumor originating from the endometrium, EC ranks first in the incidence of malignant tumors of the female reproductive system, with a trend of younger incidence [28–30]. In recent years, the extraction of effective antitumor components with low toxicity from TCM has become a new research orientation [31, 32]. DXP is attracting attention for its anticancer and immune-enhancing activities [12]. In this work, we conducted network pharmacology and cellular experiments to identify the active components, core targets, and potential mechanisms of DXP in EC.

In the present study, a total of 168 active components of DXP and 87 DXP-EC targets were screened from public databases. In the H-C-T network, we found that quercetin, associated with the largest number of targets, is the most important component in DXP for EC. Quercetin is a polyhydroxyflavonoid with various biological functions and has antioxidant, anti-inflammatory, and antiviral properties [33]. A New Jersey survey reported that lean women who consume quercetin have a lower risk of developing EC [34]. It has also been found that quercetin inhibits the proliferation of Ishikawa cells (an EC cell line) by downregulating EGF and cyclin D1 [35]. In addition, kaempferol also played a crucial role in the suppression of EC. Kaempferol is a dietary bioflavonoid with anticancer, anti-inflammatory, and antioxidant properties that inhibits human cancer cell proliferation through various mechanisms, including induction of the tumor suppressor p53 and inhibition of Er*α* [36]. Additionally, Lei's study has substantiated that kaempferol against EC by inducing cell apoptosis, arresting the cell cycle in G2/M phase, suppressing cell invasion and upregulating the m-TOR/PI3K signaling pathway [37]. These reports combined with the present research provide evidence for the therapeutic potential of quercetin and kaempferol in DXP to cure EC.

PPI analysis showed that the hub targets of DXP for EC were TP53, AKT1, INS, IL6, EGFR, CASP3, ESR1, VEGFA, MAPK3, and JUN. To annotate the functions and related pathway of 87 targets, we conducted the GO and KEGG signal pathway analysis. GO analysis results suggested that targets were mainly related to responses to oxidative stress, nutrient levels, steroid hormones, and other biological processes. The KEGG analysis results suggested that the pathways involved in DXP treating EC were mainly related to the IL-17 signaling pathway, TNF signaling pathway, PI3K-AKT signaling pathway, and ERK signaling pathway. Among them, IL-17 is a proinflammatory cytokine and is significantly expressed in a variety of cancers [38–40]. Lai's study demonstrates that IL-17 may regulate chemokines and cytokines in gynecologic cancers through activation of I*κ*B*α* and phosphorylation of extracellular signal-regulated kinase 1/2 [41]. PI3K-AKT signaling pathway is a key intracellular signaling pathway that regulates cell proliferation and cell cycle, and is closely related to tumorigenesis [42, 43]. Additionally, numerous studies have shown that the PI3K-AKT signaling pathway is involved in the malignant process of EC, such as picropodophyllin inhibiting EC cell proliferation by disrupting the PI3K-AKT signaling pathway [44]; NLRC5 promoting EC progression through activation of the PI3K/AKT pathway [45]. The ERK signaling pathway is part of the mitogen-activated protein kinase (MAPK) signaling pathway, which is involved in various cellular activities, such as cell proliferation, differentiation, apoptosis, inflammation, and other cellular activities, and is closely related to tumor-related immune escape mechanisms [46, 47]. Studies have reported that the ERK signaling pathway is one of the important pathways involved in endometrial carcinogenesis and is involved in tumor cell metabolism, growth, proliferation, survival, and angiogenesis [48].

*In vitro* experiments were performed to further verify the molecular mechanism of DXP against EC. The cell experiments demonstrated that DXP for the treatment of EC suppressed cell proliferation, arrested the cell cycle in G0/G1 phase, induced cell apoptosis, weakened the ability of cell migration, downregulated RAS expression and RAF, MEK, and ERK phosphorylation levels. These findings verified the predicted result of the network pharmacology approach.

## 5. Conclusion

In summary, we combined network pharmacology and *in vitro* experiments to verify the potential mechanism of the bioactive components and targets of DXP in treating EC. We demonstrated that DXP could inhibit the proliferation and migration of EC cells and enhance EC cell apoptosis. Moreover, DXP protects against EC malignant progression via downregulating the ERK signaling pathway. This study provides an effective drug for the treatment of EC. Meanwhile, network pharmacology analysis reveals the molecular mechanisms involved in the treatment of EC with DXP, providing a research basis for subsequent studies.

## Figures and Tables

**Table 1:** Active components of DXP.

Chemical_name	QED	Chemical_name	QED
Beta-elemene	0.5799	Phosphatidic acid	0.4043
Phenylalanine	0.5664	Glabrone	0.8364
Scopoletin	0.5425	Echinatin	0.6610
Guercetol	0.5064	Glabrol	0.5836
Quercetin	0.5064	Nonanoic acid	0.5775
Ursolic acid	0.4433	NON	0.5672
Calycosin	0.8850	(-)-Catechin	0.5139
Formononetin	0.8529	Gancaonin L	0.4938
Glabridin	0.8323	Hemo-sol	0.4838
Chrysin	0.8206	d-limonene	0.4838
Eugenol	0.6955	Alpha-limonene	0.4838
Thymol	0.6510	Stearic acid	0.3017
Kaempferol	0.6372	Catechin	0.5139
Farnesol	0.6157	WLN: QV4	0.5826
isoliquiritigenin	0.5824	hexanoic acid	0.5687
Scopoletol	0.5425	Pentadecylic acid	0.4059
Capsaicin	0.5370	Myristicin	0.6887
IPH	0.5172	Scatole	0.4871
Puerarin	0.4049	Coumarin	0.4124
Glucuronic acid	0.3250	PAC	0.6684
Z-ligustilide	0.6667	Caprylic acid	0.5818
Linalool	0.6172	Octanoic acid	0.5818
Salicylic acid	0.6129	Oleanolic acid	0.4460
BUA	0.5445	Anethole	0.6262
L-Carvone	0.5247	Vanillin	0.5173
BuOH	0.5171	Gallocatechin	0.4426
Adenine	0.5125	WLN: VHR	0.4967
Crocetin	0.5030	Isorhamnetin	0.6678
Ethanol	0.4049	Geraniol	0.6172
IFP	0.3920	Mairin	0.4635
glutamate	0.3835	GLB	0.3046
Palmitic acid	0.3653	Histidine	0.4184
Esculetin	0.3579	EUG	0.6993
LPG	0.3562	HEX	0.4628
Alanine	0.3562	Vitexin	0.3497
Deoxygomisin a	0.7145	Methyleugenol	0.6599
Butylated hydroxytoluene	0.7531	EB	0.5161
Apocynin	0.6736	2-Heptanone	0.5103
Paeonol	0.5478	Alpha-glycyrrhetinic acid	0.5188
Alpha-humulene	0.4851	Naphthalene	0.5114
Stigmasterol	0.4599	OYA	0.3958
Hyacinthin	0.4290	DFV	0.8226
Acetic acid	0.4199	Liquiritigenin	0.8226
Baicalin	0.3617	WLN: QVR BVQ	0.6891
Isovitexin	0.3497	1,2-Benzenedicarboxylic acid	0.6891
Choline	0.3405	Licocoumarone	0.5799
Tetradecane	0.3217	Glabranin	0.8174
Caffeic acid	0.4750	PHPH	0.5905
Pinocembrin	0.8226	Genkwanin	0.8862
PHB	0.6129	5,4′-Dihydroxy-7-methoxyflavone	0.8862
5-Indolol	0.5651	PLO	0.7502
3,4,5-Trihydroxybenzoic acid	0.4656	O-xylene	0.4767
Gallic acid	0.4656	Angelicin	0.4670
Umbelliferone	0.3628	Dodekan	0.3809
Morusin	0.6040	Dodecane	0.3809
DTY	0.5110	Epigallocatechin	0.4426
Genipin	0.5093	Beta-glycyrrhetinic acid	0.5188
Licochalcone A	0.4563	Liquiritin	0.4838
Beta-sitosterol	0.4354	Guasol	0.5771
Angelicin	0.4354	Guaiacol	0.5771
Zoomaric acid	0.3256	BU3	0.4701
Uralenic acid	0.6249	PYG	0.4560
18Beta-glycyrrhetinic acid	0.5188	Isoeugenol	0.7273
Glycyrrhetinic acid	0.5188	Ethyl protocatechuate	0.5494
18Alpha-glycyrrhetic acid	0.5188	Safrol	0.6221
DBP	0.4752	Malic acid	0.4307
Myristic acid	0.4490	Fraxetin	0.4141
Fructose	0.3101	PCR	0.5390
DEP	0.6925	P-cresol	0.5390
Py	0.4453	O-thymol	0.6510
FER	0.7180	Carvacrol	0.6510
Isoimperatorin	0.4856	Licochalcone B	0.4567
Lauric acid	0.3925	Methylglyoxal	0.3144
Alpha-linolenic acid	0.3326	ICO	0.6690
Succinic acid	0.5303	Hexenal	0.3885
PHA	0.5664	Adonitol	0.3082
L-Ile	0.4718	Butal	0.4435
h-Met-h	0.4506	WLN: VH6	0.3957
L-valine	0.4266	Nonanal	0.3938
Istidina	0.4207	Decanal	0.3868
L-valin	0.4120	PTL	0.3745
Prolinum	0.3867	Trans-2-nonenal	0.3144
Glutamine	0.3835	2,4-Heptdienal	0.3076
Protocatechuic acid	0.5265	Prunetin	0.8850

**Table 2:** Enrichment signaling pathways of KEGG.

ID	Description	*P*.adjust	Count
hsa05215	Prostate cancer	6.24*E* − 30	27
hsa04933	AGE-RAGE signaling pathway in diabetic complications	3.62*E* − 25	24
hsa01522	Endocrine resistance	5.68*E* − 24	23
hsa05205	Proteoglycans in cancer	7.11*E* − 19	25
hsa05219	Bladder cancer	9.54*E* − 19	15
hsa01521	EGFR tyrosine kinase inhibitor resistance	1.86*E* − 18	18
hsa05212	Pancreatic cancer	2.61*E* − 17	17
hsa05225	Hepatocellular carcinoma	2.61*E* − 17	22
hsa05224	Breast cancer	2.61*E* − 17	21
hsa04657	IL-17 signaling pathway	3.59*E* − 17	18
hsa04917	Prolactin signaling pathway	1.39*E* − 16	16
hsa05213	Endometrial cancer	1.94*E* − 16	15
hsa05214	Glioma	4.10*E* − 16	16
hsa04668	TNF signaling pathway	7.33*E* − 16	24
hsa04210	Apoptosis	1.32*E* − 15	18

## Data Availability

The data used to support the findings of this study are available from the corresponding author upon request.
